# Wellbeing in Brass Bands: The Benefits and Challenges of Group Music Making

**DOI:** 10.3389/fpsyg.2019.01176

**Published:** 2019-06-11

**Authors:** Victoria J. Williamson, Michael Bonshor

**Affiliations:** Department of Music, University of Sheffield, Sheffield, United Kingdom

**Keywords:** wellbeing, health, group music making, performance, survey, brass bands

## Abstract

The wellbeing impacts of group music making have been established by evidence-based research. However, studies have largely focused on one group music activity; singing in choirs. To what extent can these wellbeing impacts be considered representative of group music making? This paper presents a survey of wellbeing impacts in brass band players. A wellbeing survey was designed to obtain qualitative information as well as quantitative data for computing descriptive statistics regarding both positive and negative impacts of group music making on wellbeing. The survey was distributed via Brass Bands England and 346 adult brass band players reported self-perceived wellbeing impacts across five categories; physical, psychological, social, emotional, and spiritual. Responses were analyzed through a descriptive statistical approach combined with an applied thematic analysis that identified the wellbeing impacts expressed by the performers, as well as their valence (positive vs. negative). Areas of overlap between choral practice and brass band work were identified, most notably in the categories of physical, psychological, and social wellbeing; enhanced respiratory function and body posture, reduced stress, improved general mental health, and regular social interaction. We also identified wellbeing themes that are less common in choral research; impacts relating to the brass bands' physical demands, competitive tradition, community roles, and cross-generational social structures. Based on findings, we created a visual model of group music making impacts across five wellbeing categories as a basis for future research. A wider appreciation of the relationships between group music making and wellbeing can be achieved by expanding the present research base to varied music ensembles and adapting the present model to emerging findings. Testing in this systematic way would enhance understanding of the general wellbeing impacts of group music making that might be accounted for by universal brain and body processes vs. wellbeing impacts that may be unique to different ensemble types due to their particular performance styles, practice demands and traditions.

## Introduction

Wellbeing is an aspirational term relating to our state of health and happiness. As a concept it has many definitions across research and government policy (Pollard and Lee, 2003), a situation that has raised difficulties when it comes to measuring wellbeing, interpreting data, and implementing/evaluating interventions. Dodge et al. (2012) proposed a definition to capture the multifaceted nature of wellbeing with the aim of aiding research and policy to advance with a unified concept.

“*Stable wellbeing is when individuals have the psychological, social and physical resources they need to meet a particular psychological, social and/or physical challenge.”* (p. 337)

Making music in a group is one means by which people can enhance wellbeing in the terms outlined above, as it has been associated with improvements in physical and mental health, as well as community and society wellness (DeNora, 2013; MacDonald et al., 2013; Ansdell and DeNora, 2016). However, research into group music making has tended to focus on one form: choirs (Clift et al., 2008). One explanation for this situation is that choral studies, in of themselves, are so diverse in focus, methods, sample characteristics, data, and analysis, that providing a coherent synthesis of the evidence linking this music activity to wellbeing presents a significant enough challenge (Clift and Hancox, 2010). Indeed, further links between choral participation and wellbeing are constantly emerging (Moss et al., 2017). A small body of research has explored wellbeing in other groups, such as drumming circles (Fancourt et al., 2016; Perkins et al., 2016; Ascenso et al., 2018) and orchestras (Kenny et al., 2014). However, these studies are substantially fewer in number, relatively small in population size, and utilize highly individual methods, including population survey, focus groups and psychobiological assessment. Given the challenge presented by the diversity in choral research alone, it is prudent to limit our literature review on the impacts of group music making for wellbeing to choirs so that the present study may provide a directed contribution to the majority literature.

The overall conclusion from choral research is that time spent in a music group is linked to enhanced wellbeing for the majority of people irrespective of age, gender, nationality, or baseline wellbeing status (Beck et al., 2000; Livesey et al., 2012; Mellor, 2013). We aim to understand whether the kinds of wellbeing impacts that have been reported in these studies are also found in a different form of so far largely unexplored group music making: brass banding. In keeping with the definition of wellbeing provided above, we review choral wellbeing findings concerning the physical, psychological and social impacts of group music making. It is not in the remit of the present work to define or delineate the multiple, complex connections between these themes or speculate regarding the levels on which they operate. Rather, in reviewing the evidence our aim is to determine a set of established wellbeing impacts of group music making that have received broad agreement and replication in the literature.

Improved physical wellbeing is a firmly established impact of group music making and is amongst the simplest to define since studies can verify self-report with quantifiable measures related to bodily function and state of health. Creech et al. (2013) is one of many studies to report that a range of health problems were reduced in individuals who took part in group music making. Areas of self-reported physical health that have been replicated in relation to choir practice include improvements in respiration, body posture, and muscle tone (Bithell, 2014). A slower respiration rate can have a soothing effect on choristers in the short-term and can be beneficial for cardiovascular function in the longer term (Vickhoff et al., 2013). Synchronized breathing can lead to entrainment of heart beats, which has been linked to external behavioral manifestations, such as increased co-operation between members (Vickhoff et al., 2013).

Experimental studies have demonstrated the body and brain mechanisms that underlie physiological responses to choir practice. This includes increases in salivary immunoglobulin A, an important antibody agent within the immune system (Kuhn, 2002; Kreutz et al., 2004), and reductions in the stress hormone cortisol (Beck et al., 2000). Pain thresholds can increase following choral practice, a finding has been interpreted as a proxy measure for the release of β-endorphins in the body (Pearce et al., 2015). There have also been reports of increased oxytocin, which is known to have a positive impact on prosocial behaviors, such as bonding, developing trust and group solidarity (Grape et al., 2002).

Physical benefits of choral practice have been particularly well evidenced in studies of older people (Cohen et al., 2006), where choristers report higher ratings of physical health, fewer health problems, fewer falls leading to physical injury, and a reduction in the need for daily self-dose medications, such as over the counter painkillers. This research has been extended to the therapeutic applications of singing for adults with physical health problems (Eades and O'Connor, 2008; Dingle et al., 2013). Group singing can result in improved respiration and speech quality in Parkinson's disease (Di Benedetto et al., 2009), and has physical benefits for patients with respiratory problems, such as emphysema (Engen, 2005), asthma (Irons et al., 2010) and chronic obstructive pulmonary disease (Bonilha et al., 2009). In sum, it has been concluded that “*singing exercises the lungs and heart, tones the abdominal and intercostal muscles, increases oxygenation of the blood, which in turn increase mental alertness, improves stamina posture, and procures the much sought after feel-good factor, popularly related to the release of endorphins”* (Bithell, 2014: p. 237).

Evidence of psychological wellbeing is more difficult to capture compared to physical wellbeing; definitions often vary and measurement more frequently relies on self-report questionnaires. Despite these challenges, the impact of choral practice on psychological wellbeing is well acknowledged (Unwin et al., 2002; Judd and Pooley, 2014) and is discussed largely in relation to the concept of eudaimonic wellbeing. This term describes the degree to which a person feels they are “fully functioning” psychologically and not simply deriving short-term experiences of pleasure (hedonic wellbeing: Deci and Ryan, 2001, 2008). A reduction in self-perceived anxiety and stress following group singing is an outcome that emerges across choirs of varying ages and backgrounds (Bailey and Davidson, 2003), as are increases in sense of achievement, self-esteem (Bailey and Davidson, 2002, 2005), and mindfulness (Lynch and Wilson, 2018).

Cognitive engagement is a psychological impact that has been linked to group activities where there is an element of learning, especially as we age (Jenkins and Mostafa, 2015). In choirs, cognitive engagement is inherent to the task at hand thanks to music-specific demands, such as learning new singing techniques or music to be performed. Engagement with these challenges increases an individual's focus of attention, at least at the time of the activity. This marshaling of focus can help direct attention away from life events that have negative physical or mental consequences for the individual, a process that has been linked directly to an increased sense of achievement in relation to goal attainment (Clift and Hancox, 2001, 2010; Bannan and Montgomery-Smith, 2008).

Cognitive engagement is not a linear positive continuum, but one where an individual seeks an optimum level for maximizing their state of wellbeing. Too little engagement is linked to boredom whereas too much can be experienced as stress (Keller et al., 2011). The objective is the achievement of “flow” (Csikszentmihalyi, 2002), an optimum state of performance that occurs when people participate in an activity of high interest to them. Flow is characterized by high concentration leading to a loss of the sense of passing time and of self-consciousness, an increase in self-confidence, and temporary distraction from both internal and external concerns. The fact that flow can be derived from group music making explains why group singing activities have been found to be beneficial even in non-performing choirs (Bithell, 2014). Csikszentmihalyi (2002) drew particular attention to the power of group music participation in generating flow stating: “*Singing in a choir and playing in an amateur string ensemble are two of the most exhilarating ways to experience the blending of one's skills with others”* (p. 112)

Finally, in the triad of wellbeing categories defined above, there are the social benefits. This aspect of wellbeing highlights the additional benefits that are offered from participation in choirs in relation to the social dimension of group music making, in contrast to those wellbeing benefits that may at least partly be ascribed just to the act of singing. The availability of a community is important to wellbeing because human beings are social animals to such an extent that isolation and loneliness are associated with serious wellbeing detriments including an increased risk of mortality (Holt-Lunstad et al., 2015). Singing groups help tackle loneliness and isolation in older people (Cohen et al., 2006), people who are affected by homelessness (Bailey and Davidson, 2002), those living with mental health problems (Dingle et al., 2013) and people who have spent time in prison (Silber, 2005; Cohen, 2009).

Choristers report that the inherent collaboration, co-operation and team spirit of the group activity can increase their sense of community (Bailey and Davidson, 2002; Bonshor M., 2016). Choir membership and the presence of peer role models can provide an individual with a sense of group identity (Bonshor, 2017a). Furthermore, a choir can provide a structure within which an individual can experience a consistent sense of social cohesion, trust and reciprocity (Faulkner and Davidson, 2006; Parker, 2010; Dingle et al., 2013), a concept that has been discussed in choral research in relation to an individual's “social capital” (Livesey et al., 2012).

Social bonding may be particularly effective within singing groups as compared to other group activities. Pearce et al. (2015) compared social bonding experiences of three classes over a 7 months period; singing, crafts and creative writing. By the end of the courses, all three classes were associated with increased social bonding, but the singing class bonded earliest. This accelerated bonding effect was ascribed to a higher sense of “entitativity” in the music group, the idea that the group exists almost immediately as a meaningful social unit or “coherent whole” (see Stewart and Lonsdale, 2016). This concept was variously ascribed to the presence of team-based activities with shared short-term goals, to the greater need for physical synchronicity (Kirschner and Tomasello, 2010), and to the kind of physiological and psychological effects outlined earlier. Another consideration is the role of group-based (i.e., collective) positive feedback in both verbal and body language terms that occurs frequently during musical practice (Bonshor, 2014).

The triad of physical, psychological and social wellbeing themes outlined above mirrors the definition of wellness used by the World Health Organization (1946). However, there are benefits to considering additional terms relating to wellbeing impacts, since “psychological” benefits in particular can often come across as a nebulous concept in a self-report scenario. Clift and Hancox (2001) identified six dimensions of wellbeing that could be attributed to choral singing. Four of them are akin to themes from the wellbeing triad (relaxation, breathing and posture, heart and immune system, and social benefits). The remaining two categories offer opportunities for additional insights using concrete, highly relatable terms: “emotional” and “spiritual” benefits. Emotional benefits in terms of mood and moral support alongside reductions in emotional symptoms of depression are common themes in the choral literature (Bailey and Davidson, 2005; Myskja and Nord, 2008; Creech et al., 2013); spiritual benefits are less well represented to date. These two categories were added to physical, psychological, and social in our study in order to obtain the broadest possible insights.

The present review of choral singing impacts has identified two issues regarding wellbeing in group music making; a need to expand current hypotheses beyond singing and a need to review both positive and negative impacts. The current study meets the first issue through an open examination of the wellbeing impacts perceived by brass band ensemble players. The aim is to outline reported wellbeing effects across five categories (physical, psychological, social, emotional, and spiritual) and to contrast them with findings from choral research. Specifically, we examined the extent to which the major themes identified in the above literature overlap with those that emerged from our own thematic analysis. This design allows for the potential to uncover candidates for universal impacts of group music making on wellbeing, which may be tested in future studies, vs. wellbeing effects that are likely to be specific to particular group music making practices, which in the present case may help inform occupational health practices within brass banding.

The second general issue in choral research has been the focus on positive wellbeing. Many studies use open questionnaires however, the questions are often cued toward positive impacts (Beck et al., 2000; Clift and Hancox, 2001). Kreutz and Brünger (2012) is an exception, a study that offered a clear focus on negative wellbeing impacts of group music making. In total, 24.7% of their sample reported negative experiences associated with choral singing that included poor communication or leadership, social problems (marginalization, unacceptable behavior), lack of perceived musical competence in self or others, hurtful critique, and on rare occasion issues with vocal, physical or mental health. Whilst this study is not the only one to report negative findings (see Livesey et al., 2012; Bonshor, 2014, 2017b), the detail in this report motivates an aspect of the present study; the use of an open questionnaire that avoids leading people to report only the positive impacts of their brass band practice. Our aim is to derive a balanced, holistic overview of wellbeing effects in brass band players, as modeled by research into choral singing (Clift and Hancox, 2010). To capture the balance of positive and negative impacts in the present study reports, the valence of comments will be subjected to a second independent analysis.

There is little literature on wellbeing in brass bands from which we may draw expectations for this study. One exception is a small study by Lowe (2013), who set out an evolutionary biology argument for wellbeing effects in brass bands based on Boyden's (2004) list of intangible wellness needs. This includes opportunities for creative behaviors, and an environment conducive to a sense of personal involvement, purpose, belonging, responsibility, challenge, self-fulfillment, and comradeship. The list of positive factors provided the framework for Lowe's survey of 21 brass band members, which comprised 40 Likert scale statements. Factor analysis confirmed the existence of three categories; personal, communal and task-specific musical factors. This result confirms the importance of psychological impacts as seen in choir studies, namely short-term goal success, challenge, and self-fulfillment, as well as the social elements of group music making based on a sense of belonging and shared sense of purpose. It also highlights the importance of music-specific impacts, in particular in relation to the contesting tradition within brass band history and culture. Overall, the Lowe (2013) study motivates a larger study of brass band players to reveal the full extent of the wellbeing impacts and to gather information for the first time in this population on both positive and negative effects.

Like Lowe (2013), the present study utilizes a survey instrument as the ideal method to gather data regarding personal experiences when their breadth and depth is uncertain. This is the case in the present study since no research to date has approached the question of wellbeing in brass bands using open questions across general categories using a holistic perspective that allows for both positive and negative impacts. Our new survey instrument was structured based on the Dodge et al. (2012) definition of wellbeing to include physical, psychological and social impacts of music making. Three additional categories were included following Clift and Hancox (2001) with the aim of capturing the richest possible data: emotional and spiritual wellbeing, plus an open category (“other”) in order to allow for free comment.

## Materials and Methods

### The Participants

An online survey was circulated with the support of Brass Bands England (formerly British Federation of Brass Bands). Brass Bands England (BBE) has a membership of 200 bands, with ~25 members in each ensemble. BBE encouraged member participation via emails as well as their social media network. The research project was promoted on the BBE website, and “snowballing” sampling (Goodson and Sikes, 2001) was used to increase participation, as BBE members were asked to forward the survey to their personal contacts within brass banding communities outside the BBE organization.

A total of 346 participants over the age of 18 years, responded to the survey. Of these, 295 participants recorded their age (M Age = 44.8). We noted a high level of intergenerational engagement in brass band playing, with 56 participants aged 30 or under, and 56 players aged 60 or over. 75% of the survey respondents had more than 10 years' experience, with 53% of the participants having over 20 years' experience. Gender was distributed as follows: 189 male, 150 female, 2 transmasculine, 5 preferred not to specify. Among the 250 participants who indicated their instrument, the range reflected the levels of instrumentation found within a typical brass band, with cornets being most strongly represented (24%), followed by tenor horns (10%), trombones (9%) and tubas (9%).

### The Survey

The protocol was approved via the institutional ethics review board in accordance with the recommendations of the University of Sheffield's institutional guidelines. All subjects gave written informed consent in accordance with the Declaration of Helsinki. To ensure confidentiality, all survey responses were provided anonymously; any potentially identifying data (such as place names) were excluded from reports and all data was securely stored on password protected hard drives.

The online survey (Appendix 1) was created as a Google Drive document within the University of Sheffield's secure account. Demographic information about age, gender and experience of playing in brass bands, was obtained, to provide descriptive statistical data. The main research queries were framed as open text questions, with the dual aim of avoiding leading prompts that risk a “demand response” (Robson, 1993) whilst providing participants with an opportunity to describe their experiences in their own words. In this way, it was intended that there would be space for respondents to write openly about both positive and negative impacts upon their wellbeing, and for the emergence of effects that had not been anticipated by the researchers in advance of the project.

The main survey question asked “*How does brass banding affect your life?”*, with space for either positive or negative responses related to five specific categories of wellbeing, namely physical, psychological, social, emotional and spiritual wellbeing. For this open question, it was made clear that there was an option for participants to state that there was “no effect for me.” This was to ensure that participants were not tempted to “manufacture an opinion for the survey” (Robson, 1993: p. 248). A final question asked participants to add information regarding any other ways in which they felt that brass band playing had affected their wellbeing that were not covered by the five categories.

### Analysis

An applied thematic qualitative analysis protocol was applied to examine systematically the themes expressed by the brass band players across each wellbeing category. Applied Thematic Analysis (Braun and Clarke, 2006) is a methodological framework for qualitative text analysis that requires a systematic protocol where any assertions are firmly grounded within the data. The form of the data drives choices regarding the structure of both the analysis and the presentation of outcomes. Here, the analysis protocol focused on: (i) summarizing the main themes within each wellbeing category as expressed in quote segments, (ii) drawing a clear division by valence of the reports, i.e., between positive and negative wellbeing effects, as well as those that represent a mixture of two valences within the same quotation. These two stages of analysis, coding themes and valence, are detailed below.

#### Coding Themes

##### Preparing the data

The wellbeing categories within the questionnaire were physical, psychological, social, emotional, spiritual, and other. The “other” category was subjected to a preliminary stage of analysis to determine the extent to which people used this prompt to replicate statements from the five other categories or provide more detail in relation to them. Simple replications by the same individual were deleted from the dataset to avoid “double counting.” Statements that provided additional detail that clearly related to one of the five main categories were migrated to their respective columns within the dataset. Following this process there were no remaining unique statements within the “other” category; hence it was determined that the five categories of physical, psychological, social, emotional and spiritual wellbeing were adequate to capture the wellbeing statements from the brass band members.

Once the data were prepared in the manner above, attention turned to the detail within the statements. Firstly, each statement was segmented where necessary. If the statement was short (i.e., a few words or a short sentence) with one main wellbeing category clearly divisible, then it was left in the category assigned by the participant. If the statement contained reference to more than one of the five wellbeing categories (e.g., physical and social impacts mentioned under the prompt “physical”) then the material was segmented and part-migrated so the relevant aspects of the statement would fall under the relevant wellbeing categories. This resulted in a list of quotes (whole or segmented) under each of the five wellbeing categories that could then be subjected to thematic analysis.

##### Thematic analysis

The data coding followed a simplified version of the systematic qualitative thematic analysis technique based on Braun and Clarke (2006) that was outlined in Williamson et al. (2014). This form of explorative thematic analysis provides a way to code quotes from text responses that prioritizes the minimization of subjective bias.

The protocol was simplified so that one coder completed the line by line coding stages. This decision was made due to the subject experience difference between the two coders. The second author is a highly experienced music educator who specializes in vocal training and is trained in brass band instruments. As the “expert” coder (i.e., in brass band practice), they conducted the initial data analysis steps to ensure that the participants' views were most accurately interpreted in performance context. Validity of the expert coder's work was ensured to the highest standard possible by instigating a protocol whereby they were consistent and coherent in their coding (detailed below). The second (non-expert) coder joined the analysis at two stages to further ensure validity of the coding protocol.

During stage one, each quote was read line-by-line by the expert coder and the text within was coded into themes, short words or phrases that represented the core expression. Each quote could contain one or more themes depending on length and density of ideas. As theme coding continued the expert coder kept extensive notes on the nature of their translation, any emergent relationships and hierarchies between themes and potential subthemes. Where appropriate themes were replicated in order to establish patterns of frequency. By the end of this first step in the process a theme codebook had been created with all the themes and subthemes listed.

For step two in the analysis, definitions were created for each theme and subtheme within the codebook. At this point the two coders (authors) met in person and discussed the emergent codebook and its definitions, with constant reference back to the original quotations for justification. This process completed a working theme codebook that represented the data in the opinion of the two coders.

For step three, the entire dataset of quotes was then re-read by the expert coder and the agreed theme codebook applied. Following this re-analysis stage, the two coders met for a final time to compare the distribution of the text into the agreed upon themes, to draw up a visual model that represented the data, and to discuss any difficulties with the codebook application. No substantial difficulties were reported in the application of the codebook, though a number of the theme labels were shortened at this stage to allow for a comprehensive visual diagram.

#### Coding Valence

For this second analysis, both authors independently coded all quotes as one of four valences; positive (+), negative (-), or a mixture if the statement presented both a positive and a negative aspect (+/– or –/+, depending on the order of the shifting valence). Both coders also had the option to code as U or unclear, where there was insufficient evidence within the quotation to be clear on the Valence expressed by the author (e.g., the quote simply read “Breathing”). Where there was disagreement between the coders following the double coding, the ambiguous quotes were removed from the database before the final calculation of valence distribution by wellbeing category as presented in the visual model (as shown in Table 1). The nature of the disagreements regarding valence assignment are detailed in Appendix 2. This process led to the identification of a clear outline of positive and negative, as well as mixed, wellbeing impacts across a broad range of wellbeing categories.

**Table 1:** Frequency of code occurrence for the wellbeing themes.

**Wellbeing category**	**Quotes coded for theme**	**Number of disagreements on valence**	**Quotes coded for valence**	**Percentage positive valence**	**Percentage negative valence**	**Percentage mixed valence**
Physical	409	15	394	82.23%	17.51%	0.26%
Psychological	401	5	396	94.70%	3.54%	1.76%
Social	548	4	544	96.51%	3.31%	0.18%
Emotional	245	19	226	81.4%	3.6%	15.0%
Spiritual	55	1	54	94.5%	5.5%	0%

*Percentages are adjusted to reflect removal of statements where the valence was ambiguous, i.e., could not be determined via independent coding of two reviewers*.

## Results

### Wellbeing Themes and Their Valence

In total, 1,658 individual quotes were coded. Table 1 details the number of quotes that were coded in each of the wellbeing categories. The next column reports the number of quotes where the two coders did not agree on valence (Appendix 2 for detail). In summary, the majority of disagreement (56.81% of occasions) occurred when one coder applied a valence label (positive or negative) and the other coder marked the quotation as Unclear, as they did not feel there was sufficient evidence from the words alone to be sure of the expressed valence. This double coder stage was analyzed using Cohen's Kappa, resulting in an inter-rater agreement level of 0.871 (SE = 0.018, 95% CI = 0.836–0.907), a strength of agreement considered to be “very good”. Following the removal of all valence ambiguous materials, the fourth column in Table 1 shows the number of statements that were coded positive, negative or mixed in valence.

Following the removal of unclear codes from the dataset, 89.87% of quotes (mean average) were positive, 6.69% of quotes were negative, and 3.44% represented mixed valence expressed within the same statement. The vast majority of mixed valence statements were in the Emotional category.

**Figure 1:**
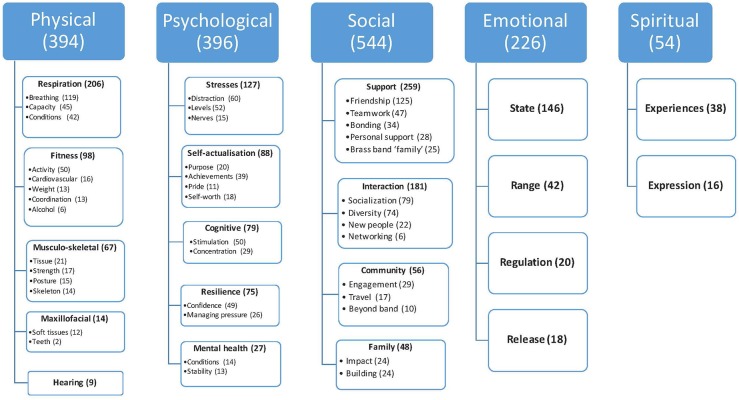
Visual depiction of the five wellbeing categories and their emergent themes. Themes are ordered in terms of their descending relative size as given by the number of quotations. Numbers represent number of quotations where valence was agreed by both researchers, to allow for consistency with Table 1.

Figure 1 presents a visual model of the emergent themes within each of the five wellbeing categories. The model delineates the relationships between the theme groups in a unified and holistic way, following the valence analysis (i.e., disagreements shown in column two of Table 1 are removed from the totals shown in Figure 1). The next section details the evidence that supports the depicted structure.

#### Evidence

Below we provide text evidence for the visual model in the form of quotations provided in response to the open questions under the five wellbeing categories. Each category is shown as indented bold text, followed by bold terms representing the emergent main themes. The sub-themes that emerged within each theme are listed in normal font. In each case the number of text segments coded is noted in brackets (excluding examples where valence disagreed), followed by a breakdown by valence category. As in Figure 1, themes are listed in order of descending size according to the total number of quotations received. An exemplar quotation from the survey responses is given in italics to illustrate the theme from the perspective of the participants. A coding system is provided after each quote that indicates gender, years of brass band ensemble experience, and age. A “U” is substituted where demographic details were not provided.

**Physical (394: 324 positive, 69 negative and 1 mixed)**: Comprises five main themes and fourteen subthemes.

**Respiration (206: 203 positive and 3 negative):** Comments attributing changes in respiratory function to brass banding. “*As the important part of playing is controlled breathing I feel it must be a help to keeping your lungs healthy*.” M.20.68
Breathing (119: 117 positive and 2 negative): Comments on general effects on respiration. “*Helps with breathing and breath control, and I am more conscious of my breathing and I remember to breathe more deeply*.” F.2.47
Capacity (45 positive): Comments relating to ability to take deep breaths and play long phrases. “*Lung capacity impresses NHS staff when measured for non-banding purposes*!” M.20.57
Conditions (42: 41 positive and 1 negative): The person reports changes in respiratory illness conditions including but not limited to asthma, pneumonia, chest infections, and chronic heart failure. “*I'm asthmatic and it has helped me gain a great deal of control over my breathing (despite being incredibly unfit and overweight I come out as ‘elite sportsman' when I have my breathing monitored by my doctor)*.” M.20.U
**Fitness (98: 70 positive and 28 negative):** Comments related to an impact on general fitness, weight or level of physical activity that participants attribute to their brass banding. “*A 2-hour rehearsal or concert is more invigorating than a workout in the gym*.” M.20.U
Activity (50: 41 positive and 9 negative): Comments related to physical activity levels, including increased stamina. “*Exercise is not limited to breathing and playing music […] Arranging chairs, shifting instruments into venues etc also contribute to keeping me fit and active.”* M.2.71
Cardiovascular (16: 15 positive and 1 negative): Comments related to cardiac function, including heart rate and blood pressure. “*Tremendous cardio-vascular system. Resting pulse of 60 and bp 126/75 and I'm 62 who drinks too much red wine!”* M.20.62
Weight (13: 1 positive and 12 negative): Weight gain was a concern for some participants, who attributed this to extended periods of sitting, rehearsal time limiting opportunities to participate in sporting activities, and the increased alcohol intake and unhealthy eating which some players reported as part of brass band “culture.” “*The social side of banding has meant a lot of beer and curries, so I am no longer in ‘prime shape.' Probably better described as morbidly obese*.” M.20.37
Co-ordination (13 positive): Comments related to hand to eye co-ordination and motor skills. “*It also helps with co-ordination—fingers, lips, muscle, brain.”* M.20.U
Alcohol (6 negative): Reported increased intake of alcohol. “*There is something of a drinking culture within some bands which can be destructive*.” F.20.54
**Musculo-skeletal (67: 44 positive, 22 negative and 1 mixed):** Comments related to alterations in movement and flexibility. Includes references to hand to eye co-ordination, manual dexterity or flexibility, and ongoing musculo-skeletal issues. “*I suffer from Rheumatoid Arthritis—I believe playing an instrument helps keep my joints a little more flexible*.” F.6.46
Tissue (21: 7 positive and 14 negative): Comments related to impact on muscles and soft tissues. Whilst some players reported “better abdominal muscles” and “improved muscle tone,” others reported “muscle strain.” “*A consultant suggested that my neck and back problems may be due to over strong muscles in certain areas due to holding an instrument for prolonged time.”* M.20.47
Strength (17: 15 positive, 1 negative and 1 mixed): Comments related to core and upper body strength. “*I play one of the bigger instruments so I'm definitely stronger than I would have been if I didn't play the tuba*.” F.11.22
Posture (15 positive): Comments related to comfort and posture when standing and sitting. “*Helps you to have good posture*.” F.20.U
Skeleton (14: 7 positive and 7 negative): Comments related to positive effects on joints and bones, such as keeping “*fingers moving and the joints supple*.” F.11.28.
**Maxillofacial (14: 6 positive and 8 negative):** Comments relating to the function and health of the lips or teeth due to the continued physical pressure of playing brass instruments. “*Cold sores [… and] may have contributed to loose teeth!”* M.20.U
Soft tissues (12: 6 positive and 6 negative): Comments relating to effects on lips and cheeks including “*stronger facial muscles, especially around the lips.”* M.2.71. “*Lip soreness when playing a lot*.” M.20.46
Teeth (2 negative): Comments relating to dental effects of brass playing, including “toothache.” “*May have contributed to loose teeth!”* M.20.U
**Hearing (9: 1 positive and 8 negative):** Comments related to hearing ability and impacts. “*I have suffered from hearing loss I feel as a result of loud percussion, this has been helped with hearing aids and I continue to play after 36 years*.” M.20.68

**Psychological (396: 375 positive, 14 negative and 7 mixed):** Comprises five main themes and fourteen subthemes.

**Stresses (127: 113 positive, 11 negative and 3 mixed):** Comments on stress levels, including brass banding as a source of “stress relief.” “*Playing in a brass band is a great therapy. Having been under considerable stress for a lot of my working life I have often come to a rehearsal or a gig feeling ‘all-in,' sometimes wishing that I needn't bother. Most often I have left afterwards feeling revived again.”* M.20.66
Distraction (60 positive): Comments describing playing as an immersive experience, which provides a distraction from stress. “*One has to concentrate on the music and in doing so abandons, if only for short periods, the everyday worries and concerns that we all experience.”* M.20.68
Levels (52: 38 positive, 11 negative and 3 mixed): Comments relating to effects upon subjective stress levels. Positive comments ranged from “reducing stress” to an “antidote to stress.” “*When stressed, going to a rehearsal calms me and helps me revalue what I was stressed about*.” F.11.24. Negative comments included stresses induced by the pressure of competing, committee work and conductor/leader behavior. “*A new musical director was appointed several months ago and in my role as secretary have found him a very difficult person to deal with and a bully. He has caused me several months of stress. I can't get the problems out of my head so I have had to resign*.” M.20.61
Nerves (15 negative): One of the most common reported stressors was performance anxiety or, in some cases, stage fright. “*Nervous shaking in pressure situations. I get very nervous in situations that I feel are ‘important performances' (e.g., playing solos, competitions). I get physical symptoms, most notably nervous shaking*.” F.20.49
**Self*-*actualization (88: 84 positive, 1 negative and 3 mixed):** Comments related to fulfilling potential and the positive feelings associated with this. “*You get a sense of achievement that can boost your self-esteem*.” F.20.U
Purpose (20 positive): Comments related to finding motivation and meaning in life. “*Has given me a sense of purpose and drive as I try to improve my playing with a group of people. I feel generally more satisfied with how I am living because I have more purpose and reason to be alive. (I only started back playing 18 months ago after a 22 years break)*.” F.11.48
Achievements (39: 37 positive, 1 negative and 1 mixed): Comments relating to achieving goals, sense of accomplishment and affirmation, and related positive feelings. “*Satisfaction & sense of achievement when I master a challenging passage of music or overcome nervous feelings when performing*.” F.20.49
Pride (11 positive): Comments on the sense of pride derived from performing well and improving. “*Pride at having progressed so far, despite starting to learn to play a brass instrument in my late 60s*.” M.2.71
Self-worth (18: 16 positive and 2 mixed): Participants reported changes in their self-worth and self-esteem due to their evolving identity as a musician. “*Nurturing and developing your own musical talent leads to many accomplishments that boost your self-esteem over time.”* M.11.26
**Cognitive (79 positive):** Benefits related to cognitive functions including mental alertness and agility. “*I think my brain function aging is being slowed by my musical activities, concentration and coordination of mind/body is being preserved*.” M.2.71
Engagement (50 positive): Benefits derived from cognitive engagement, including keeping an active mind, improving the ability to learn, and enhancing memory. “*Having now played for nearly 60 years, reading and playing challenging music is not only fun but helps to keep my brain active and functioning well*.” M.20.68
Concentration (29 positive): Improvements in concentration and associated benefits. “*I find playing music, having to concentrate and follow a conductor, a wonderfully cleansing psychological experience. Focusing 100% of your concentration on one thing can be like a meditation at times and feels very healthy and beneficial*.” M.2.33
**Resilience (75: 73 positive, 1 negative and 1 mixed):** Comments related to resilience include “persistence,” “determination,” “becoming a stronger person,” learning to “deal with success and failure,” becoming better at “coping with stress” and “pressure,” and “coping with nerves.” “*Feeling happy, occupied and fulfilled leads to better mental health and increased resilience when dealing with difficult things. Banding gives me a safety net to protect against adversity.”* F.11.49
Confidence (49: 47 positive, 1 negative and 1 mixed): Shifts in confidence levels, which were transferable beyond the band room. “*I've become more confident since playing with a band, I think owing to the fact that playing on stage in front of an audience demands a certain level of confidence and everyday life seems like no pressure in comparis*on.” M.2.23
Managing pressure (26 positive): Comments relating to learning to deal with stress, performance “nerves,” and “pressure situations”. “*I feel that Contesting has made me mentally tougher. I feel that I can approach stressful performances/situations with more positivity. I have found through 20*+ *years of banding at all levels that I can rise to the challenge of a contest. Which has helped me dealing with other aspects of real life!”* F.20.38
**Mental health (27: 26 positive and 1 negative):** Comments relating to effects on self-perceived psychological stability and changes in mental health conditions. “*Having suffered (& occasionally still suffering) from depression and anxiety I can confirm that playing in a brass band has helped me through several difficult patches in my life.”* M.11.U
Conditions (14: 13 positive and 1 negative): Comments regarding mental health conditions, including anxiety and depression. “*The one activity I have been able to maintain and enjoy during bouts of depression*.” F.11.40
Stability (13 positive): Comments related to maintaining an even temperament and sustaining general mental wellbeing. “*Keeps me sane*.” F.20.52

**Social (544: 525 positive, 18 negative, and 1 mixed)** Comprises four main themes and thirteen subthemes.

**Support (259 positive):** Comments relating to practical and emotional support networks. “*We look after each other and are there to support each other at all times. It is wonderful to know that there is always a friendly, caring person who will listen and give support.”* F.2.20
Friendship (125 positive): Comments relating to personal, mutually supportive relationships between players, which often extend beyond the music-oriented activities. “*My band is very social and form our main group of friends. Also the banding community is very friendly and social so its lovely meeting up with bandsmen you haven't seen for a while*.” F.20.33
Teamwork (47 positive): Comments related to task-oriented relationships arising from working together toward shared goals. “*The social effects from being part of a team working to produce a good performance which leads to a feeling of joy when that point is reached*.” M.20.68
Bonding (34 positive): Comments related to affect-oriented relationships based on group cohesion and collaboration. Participants described their “sense of belonging” to a “club” or “tribe.” “*Something about breathing together, making amazing sounds together provides a bond. There is an understanding between band mates that is like no other.”* F.6.50
Personal support (28 positive): Comments related to providing and receiving interpersonal support (or conflict) in musical and non-musical contexts. “*We've shared many ups and downs in our lives and in banding. A supportive network of people*.” F.20.54
Brass band “family” (25 positive): Descriptions of the band as a “family,” in which there are close, supportive interpersonal relationships. “*The band are basically my second family so if I'm feeling crappy it's always nice to get out to see them and make some music.”* F.11.22
**Interaction (181: 177 positive, 3 negative and 1 mixed):** Comments relating to levels of social interaction as a direct result of participation in brass bands. “*For me, banding is about social interaction through music with a group of likeminded committed individuals. This creates a real sense of wellbeing*.” M.20.U
Socializing (79: 76 positive and 3 negative): Comments relating to levels of participation in social events, including musical and extra-musical activities “*If you are prepared to spend the time and effort to master a brass instrument you will never be lonely or bored again. There are so many bands out there and many are crying out for players, that you could be out every day of the week playing with some band. This in turn will lead to great social interaction with people of similar musical interests (not to mention the social pint after practice—well why waste a good thirst!!)*.” M.20.71
Diversity (74: 73 positive and 1 mixed): Comments relating to the variety of people encountered through banding activities. “*It is an opportunity to mix with a diverse range of people. I feel that growing up in a brass band environment is good for social development and helps you in other areas of your life, e.g., employment, as you are comfortable talking to people of different ages and backgrounds.”* F.20.41.
We note three sections within this theme: (1) socio*-*economic (37: 36 positive and 1 mixed) “*It is good to be with other people of differing backgrounds, abilities and views. I have always said that the brass band is social engineering”* F.20.53; (2) intergenerational (29 positive) “*I retired last year and my involvement in brass bands is now my primary interest outside my family. It also gives me contact with a wide range of people of all ages. It helps me being in contact with younger band members and assisting them to develop. I believe it also helps them to develop social skills mixing with older people and taking on commitment and responsibility.”* M.20.67; and (3) geographical (8 positive) “*In a band you will always have an instant connection with people because you share the same interests no matter where you are in the world*.” F.20.U.
New people (22 positive): Descriptions of brass band playing as an ongoing opportunity to establish new social contacts. “*It is a great way to meet people in a community and really helped me to settle in when I moved to a new area*.” F.6.24
Networking (6 positive): Descriptions of brass banding as an opportunity for developing business and professional contacts. “*Really great to help with networking- and it helped to get the job I am now in*.” F.6.24
**Community (56 positive):** Descriptions of the social benefits of interacting with the wider community, via brass band playing. “*A wider social effect—playing concerts in our community—feeling strong links with the community*.” F.20.49
Engagement (29 positive): Comments relating to contact with, participation in, and contribution to local community. “*Opportunities to contribute to community activities and charity fund raising.”* F.20.54
Travel (17 positive): Descriptions of broadening horizons through exploring new geographical locations and cultural settings: “*The opportunity to see the wider world via it, which is often subsidized by the band so people on lower incomes have the opportunity they might not otherwise get*.” F.6.39
Beyond band (10 positive): Comments relating to socializing outside band events. “*My band has an extremely social outlook and we spend many hours together both at rehearsal and other activities.”* M.20.52
**Family (48: 36 positive and 12 negative):** Comments relating to the effects on family relationships, and opportunities provided for family members via brass band membership. “*I am very happy that both my children had the advantage of growing up within the band. Band events were always a safe place for them to be and they gained a lot of their social experience within the band*.” F.20.U
Impact (24: 14 positive and 10 negative): Comments relating to the effects of brass banding playing upon family life. It can be an inclusive activity for some players “*Whole families take part in the same activity together. It binds the generations—our family has four generations of trombone players and we regularly play together.”* F.20.54. However, for others it can create division: “*It's great socially due to the people you meet but it can also have an effect on your home life if your partner isn't as understanding, hence the term brass band widows.”* M.20.47
Building (24: 22 positive and 2 negative): Comments relating the influence of brass band playing upon building family units through developing partnerships. “*I met my husband through brass banding—says it all really.”* F.20.4

**Emotional (226: 184 positive, 8 negative, and 34 mixed):** Comprises four main themes.

**State (146: 139 positive and 7 negative):** Comments relating to mood state during brass band playing, including references to pleasure, happiness, “feel good factor”/“buzz,” calm/peace. “*Band practice is an important part of my week. Playing makes me feel happy and positive*.” M.20.U. Negative feelings include disappointment, guilt and irritation about making musical mistakes. “*When things don't go well I can be very frustrated and disappointed with myself or the band*.” F.11.48
**Range (42: 8 positive and 34 mixed):** Comments on the “highs and lows” of rehearsing and performing. “*Banding is an emotional rollercoaster, especially if you compete. I regularly cycle through satisfaction, pride, sense of accomplishment, frustration, disappointment, nerves and elation. When you are so passionate about something and work hard to achieve your goals then I think it is healthy to experience all these emotions*.” F.20.33
**Regulation (20 positive):** Comments relating to mood enhancement or emotional relaxation. “*Playing music with others in a band can help lift your emotional state. It helps me to improve my mood if I am cross or down. It helps lift me and make me feel good about what I do. It is great to laugh together with others (and sometimes cry). It helps me keep on an even keel*.” M.20.54
**Release (18: 17 positive and 1 negative):** Comments related to channeling, experiencing and expressing emotions. “*I can channel distressing emotions through the instrument, therefore it is a good way of dealing with emotion without burdening others*.” F.11.30

**Spiritual (54: 51 positive and 3 negative):** Comprises two main themes.

**Experiences (38: 35 positive and 3 negative):** Comments relating to spiritual experiences during playing, which included “enhanced worship,” “spiritual benefits,” and “spiritual wellbeing.” “*There is nothing more spiritual than playing an inspiring piece of music—feeling closer to God or whoever we believe in is a happy knock on effect of playing*.” F.6.46
**Expression (16 positive):** Comments relating to active expression of spirituality during playing. “*I do understand music to be a gift from God with a spiritual dimension. It can be a means to worship and prayer and I see that my playing in a brass band can be a means to bring myself and others closer to God and also to glorifying Him in some way through a gift He has placed at my disposal*.” M.20.66

## Discussion

### Brass Band Wellbeing in Context

In the present study, we analyzed 1,658 wellbeing impact statements from 346 active brass band members for thematic content and valence. This represents the largest wellbeing-related research project within this group music-making population. Whilst the population numbers in the most commonly studied group cohort, choirs, can be larger [e.g., Clift and Hancox studied 1,124 international choral singers (2010) and 600 English choral singers (2007)], brass banding has a substantially lower recruitment rate and active performing population. A report by the Internet Bandsman's Everything Within group estimated that there were 1,234 active brass bands in the British Isles in 2018 (down from 7,321 in 1890s^1^). This compares to a 2017 census conducted by voicesnow.org.uk, which found at least 40,000 active choirs in the UK with over 2 million people singing in choirs regularly^2^. Hence our sample of brass band players is reasonable in size relative to that seen in choral research.

In line with choral research, and music wellbeing research in general, the vast majority of reports from brass band players (89.87%) related positive impacts from their group music making (Beck et al., 2000; DeNora, 2013; Mellor, 2013; Ansdell and DeNora, 2016). There are other reasons to be confident in this pattern, aside from the alignment with existing research. The survey was anonymous and online, an environment that minimizes pressure to respond in support of any agency. Furthermore, we received reports of negative impacts (around 10% when combining pure negative and mixed reports), which suggests people felt able to express a range of personal experiences and reflections. Although the level of negative reports was less than in Kreutz and Brünger (2012) (24.7%, though note this figure is based on people rather than quotes, as in our case), it was in line with Livesey et al. (2012), whose sample matched the comparatively wide demographic of the present paper. A final point relates to the experience of our participants; over half (53%) had over 20 years' brass banding experience, with five individuals having between 40 and 60 years of experience. In sum, the majority positive impacts of wellbeing reported by these 346 individuals is a result that can be taken as representative. Hence the present research helps to generalize past research showing the majority positive wellbeing impacts of group music making by extending it to a so far under researched music-making scenario.

The majority of quotes (80.82%) were in line with the wellbeing definition reported at the start of this paper, in that they consisted of physical, psychological and social impacts (Clift and Hancox, 2001; Dodge et al., 2012). The pattern of positive music-making impacts of brass banding across all the categories largely overlapped with general wellbeing benefits widely reported in choral research (detailed below) and other group music making scenarios (Kenny et al., 2014; Fancourt et al., 2016; Ascenso et al., 2018), though we noted several novel/rare wellbeing impacts to this literature that we consider here in relation to the unique experience of brass banding.

### Integration Into Current Understanding of Group Music Making Wellbeing

Following on from our visual model, we reflected back on the choral literature and identified strong overlap in wellbeing outcomes between the two forms of group music making. These were clustered around the categories of physical, psychological and social wellbeing, and match well the first four themes identified by Clift and Hancox (2001) in their model of wellbeing impacts of choral activity (relaxation, breathing and posture, heart and immune system, and social benefits). In the following sections we draw out the similarities between the groups based on the literature survey and our data, noting also the unique impacts identified in brass banding.

#### Physical Impacts

Both choral singers and band players report wellbeing impacts linked to physicality. Our participants noted similar qualitative improvements to those identified by choral singers in relation to respiration, body posture, and muscle tone (Bithell, 2014). Furthermore, we received reports relating to improved cardiovascular function (Vickhoff et al., 2013) and breathing related conditions, such as asthma and COPD, though we note that there is no conclusive medical evidence to support such self-reported improvements to serious medical conditions (Skingley et al., 2013; Lewis et al., 2016). On the last point, a number of our participants reported medical details, such as blood pressure levels and lung capacity measures. Whilst these cannot be verified from self-report, this finding offers an avenue for research wishing to expand on the breathing-related benefits of brass banding. Cooperation with doctors and access to medical records could allow for longitudinal research on people with relevant health conditions to confirm or refute the hypothesis that brass banding over long periods of time (and other high breathing-demand group music making activities) may result in measurable benefits on existing cardiovascular conditions.

An increase in physical activity is to be expected when people take on a role in choirs and brass bands, but the unique demands of brass band rehearsal and performance can be associated with wellbeing reports that are less common or absent in choral research. The first example is reports relating to maxillofacial musculature, which are uniquely challenged by the demands of brass band instruments. Here there were more negative wellbeing reports than positive (9. vs. 6), highlighting one area of physical impacts relating to pain, alongside reports of Repetitive Strain Injury and hearing issues, that should be a focus for education and occupational health in brass band organizations. A greater awareness of unique physical challenges that can occur in brass banding and the introduction of preventative/awareness training around these issues would have foreseeable positive impacts on wellbeing in this population.

Another example of a finding more common in the present population than in choral research is reports relating to improvements in upper body strength, which in brass banding was linked to transporting, performing and marching with heavy instruments. Whilst marching bands provide these additional opportunities for exercise over more static music making scenarios, the level of inactivity during extended rehearsals in non-marching bands was also a cause for some concern for some, along with the social drinking and eating that can follow rehearsals and performances. Some players felt that this combination of circumstances had led to weight gain and a lack of physical fitness.

#### Psychological Impacts

Stress management skills, self-actualization and self-esteem through goal attainment, and psychological resilience benefits made up nearly 20% of the total quotations from our sample, making these the most common psychological wellbeing impacts, as has been established in choral research (Clift and Hancox, 2001, 2010; Bailey and Davidson, 2003, 2005). Another specific reference in our data that relates to positive psychological states was the benefits of brass banding for focus and concentration of attention, with a small number of participants making reference to flow state (Csikszentmihalyi, 2002) or the positive impacts they felt following a period of time “in the zone” (Bithell, 2014). The experience of time passing in this way is also akin to that described by the term Mindfulness, defined as of a state focused attention, awareness and acceptance, and which has also been identified as a benefit of singing groups (Lynch and Wilson, 2018).

Another key psychological impact on wellbeing was the cognitive engagement provided by brass banding activity, again a strong theme of choral research (Jenkins and Mostafa, 2015). Our participants made general music-making references, such as the challenges that arises from reading and memorizing notation, ongoing monitoring of performance outcomes, and breath/body control. However, they also made references that are not typically found in choir research due to the difference in instrumentation, such as hand-body-eye coordination during marching, the difficulty of maintaining embouchure (the technique of correctly forming the mouth around the mouthpiece), and the complexity of switching to performing in different tonal keys. It is not appropriate to draw direct comparison regarding the relative difficulty of these tasks based on our data, but it is important to make the point that psychological/mental engagement challenges differ between these two group music making activities.

#### Social Impacts

Choristers have previously identified increased co-operation between members (Vickhoff et al., 2013) and team spirit (Bonshor M. J., 2016) as positive wellbeing impacts of belonging to a music making group. Brass band members also recognize these experiences (as reported in the smaller brass band survey by Lowe, 2013), with top themes in our data including “Bonding” and “Friendship,” factors that serve to combat loneliness and isolation at any age. For some participants, the unique sense of community in brass bands was labeled in a way that deserves mention. Twenty-five respondents, independent from each other (assumed from the online survey set up), referred to their “brass band family.” These members saw the band as a substitute or second family, a way of expressing the strong sense of internal community that exists within these organizations. Others also spoke of the social life that exists “Beyond band,” which ranged from dating between members through to non-music related group outings and celebrations. This informal element of brass band life has not been widely identified in choral research, though this does not mean that it is not present. The importance of this aspect of group music-making for social wellbeing implies that future research needs to look beyond the social environment of the practice/performance room to gather a full picture of how music making can foster positive social wellbeing.

Another theme that emerged in the brass band players that has rarely been noted in choral research are the intergenerational benefits of group music making. The wellbeing benefits of intergenerational engagement in singing groups has been discussed in terms of a specific project where intergenerational engagement and socialization was the focus (Varvarigou et al., 2011) however, in general choirs often rely on people from similar age groups: Moss et al. (2017) study cited an average age of 61 for their participants with a very small range. An age-related recruitment focus creates a different support dynamic and it is not our intention here to say one is better than the other. Rather, we highlight the unique benefits to wellbeing of intergenerational music making, including across families, as identified by brass band players.

Related to this finding regarding intergenerational diversity in brass bands is the importance of role models for promoting social wellbeing. Peer role models have previously been identified as an important wellbeing impact in choirs (Bonshor, 2014); the present research extends this idea to the relationship between older and younger people, the reciprocal interactions that they enjoy on a regular basis inform insights into the challenges faced by each group in daily life and destabilizes stereotypes. This is a unique view on the concept of “social capital” (Livesey et al., 2012) within group music making which highlights the benefits of ensembles that facilitate exchange between social groups that would otherwise be unlikely to interact on a regular basis outside the family unit, building trust and reciprocity.

#### Emotional and Spiritual Impacts

The final two categories of the survey were less populated compared to those above, but nevertheless offered insights into similarities between choirs and brass bands. The current respondents identified common emotional impacts of music making including positive mood states, a reduction in symptoms aligned with depression and anxiety, and the role of music making as a successful method of emotional release in relation to non-music life experiences, either through the emotional content of the music being performed or the emotional nature of performing in synchrony with a group (Clift and Hancox, 2001; Bailey and Davidson, 2002; Myskja and Nord, 2008; Creech et al., 2013).

“Range” is a theme within Emotion that is worthy of note in relation to brass bands, as it is a concept not covered extensively in choral research. This theme related strongly to the regular cycles of emotional highs and lows that brass band players experience as a result of their regular schedule of competitive performances. The theme of “Regulation” also drew upon the experience of competition in this group music making tradition, as members felt that this experience of highs and lows have given them generalizable skills in emotion regulation that they were able to apply for their wellbeing in their wider life.

The wellbeing prompt “Spiritual” allowed some brass band members to speak about the interaction between their group music making and their belief in/sense of connection to a deity, denomination or mythology, or simply a sense of closeness and belonging in their wider world. The experience of brass banding was reported to impact positively on wellbeing through providing individuals with the opportunity to enhance and extend these spiritual experiences as well as offer a unique form of expression and communication of these episodes. Though under researched at present, it is clear that spirituality is an area of wellbeing impact that needs to be included in future studies, aligning as it does with key wellbeing targets, such as “Connect” (Five Ways to Wellbeing: UK government 2008^3^ when viewed in a wider context than basic social interactions.

### Key Advances on Current Views

The first aim of this study was to determine the extent to which previous reports of wellbeing impacts from choral research could be replicated in reports from brass band players. Given the summaries above, we conclude that there is a great deal of overlap between the benefits discussed by members of both groups and hence it is reasonable to integrate the present research into a model of group music making wellbeing benefits, as we have done, that can be used by future research. A summary example of this idea of overlap can be found by turning to the quote given in the Introduction, where, based on the present evidence, the word “singing” in this quotation could be substituted justifiably for “brass banding”:

“…*singing exercises the lungs and heart, tones the abdominal and intercostal muscles, increases oxygenation of the blood, which in turn increase mental alertness, improves stamina posture, and procures the much sought after feel-good factor, popularly related to the release of endorphins”* (Bithell, 2014: p. 237).

However, we have also identified wellbeing impacts that are more strongly represented in brass bands compared to choral research. In particular, impacts relating to the unique physical demands of brass instrumentation and psychological demands of this music performance form, findings which align with the “task-specific music factors” element of wellbeing identified by Lowe (2013). Our data also related to the formal competitive tradition and community role of brass bands. In terms of the social capital of brass banding, we have drawn attention to the social exchanges that take place within this unique music-making environment and which members identify as key benefits to their wellbeing, including the strong sense of family and bonding outside the practice room, and the interactions that take place across the generations.

A second aim of our research was to introduce a simple wellbeing self-report questionnaire design that could be used by future studies of group music making. An important element of the questionnaire design, aside from the construction being based on established wellbeing definitions, was the inclusion of lesser employed categories of “emotional” and “spiritual” wellbeing in addition to physical, psychological and social elements. These were strongly indicated by research in choral practice (Clift and Hancox, 2001), a finding supported by our outcomes since we obtained 331 quotations from these categories alone. It is not typical to include “emotion” as a separate prompt in music wellbeing studies, emotions often being considered as part of psychological impacts. However, the use of this readily recognizable term as a separate prompt allowed for focus on the issue and henceforth a range of emotional impacts were reported. 13.63% of our final coded reports (after valence checks) related to emotional impacts of brass banding and 3.27% related to spiritual. We also included an open question and coded it alongside the other categories to ensure we missed no opportunities to identify wellbeing pros or cons of brass band life. The fact that the “Other” category produced no additional themes of wellbeing supports the use of these five main prompts in future research into the wellbeing impacts of group music making. As a result of this aspect of the design we confirmed the findings of Clift and Hancox (2001), that these five categories are sufficient as prompts.

As well as replicating the majority of the physical, psychological and social wellbeing benefits of choral music making in our brass band sample, we sought to provide a balanced view of impacts by inviting participants to discuss the challenges as well as the positives of making music in a group. Inspired by Kreutz and Brünger (2012) and Bonshor (2014) we opted for a general view of wellbeing effects rather than a focus on the benefits of group music making. The survey questionnaire was designed to encourage people to talk about personally experienced benefits and costs that they associated with their group music making. Providing an unbiased view of wellbeing in the manner of these reports is an important advance in the study of group music making, which has the benefit of not only widening the knowledge base relating to wellbeing impacts for future testing, but also drawing out issues that can be addressed directly with wellbeing education and awareness training for the music groups in question. Finally, we note new insights into the patterns of valence across the theme categories, in that the majority of mixed valence reports were in the “emotion” category, where people commented about the “highs and lows” of their life in brass bands. Again, this was often related to unique challenges of brass band life, such as the stresses and victories of competition and involvement in emotional events, such as Remembrance parades and other memorials, long standing community traditions, and Christmas concerts.

### Limitations on Interpretation

The present study opted for anonymous reporting so that participants were assured of confidentiality and hence felt able to express both positive and negative impacts of their brass banding activity on wellbeing. One limitation of this method is that it does not allow for triangulation of data, whereby steps are taken to assess whether the participants' views were interpreted accurately, typically be asking them if the final summaries of their comments appear valid to them. Triangulation of data could be a consideration for future studies in this area, in contexts where participants are able to report in a non-anonymous manner.

Anonymous reporting also limited the extent to which we could reliably interpret general findings. For example, the number of spiritual effect reports received was lower than that reported by Clift and Hancox (2001) and the proportion of negative reports in the present study was lower than that reported by Kreutz and Brünger (2012). There is no way to reliably interpret these differences without a way to further understand the basis for the reporting patterns within the present dataset. A triangulation protocol, such as follow-up interviews would be one way to achieve more explanatory power in future studies.

Whilst we seek to draw similarities in wellbeing impacts reported here and in other music-making scenarios, the present online survey does not allow us to understand the underlying psychophysiological or neurological reasons for the reported impacts. Wellbeing effects are often linked indirectly to the psychoneuroimmunological impacts of music making on the human body and mind, such as an increase in anti-inflammatory immune profile and sympathetic nervous system stress responses (Fancourt et al., 2016), and modulation of neurotransmitters linked to subjective experience, such as serotonin and dopamine (Altenmuller and Schlaug, 2013). We are also not able to comment on reports linking music making to body or brain function since none of our reports can be related directly to psychophysiology, without seeking further validation, and no measures of specific mechanisms, such as immune response were obtained. We have drawn associations with this research as part of our background literature as it relates to choral and other group music making scenarios, but a full understanding would require a much larger study that could incorporate hypothesis testing—could be based on the current model—alongside objective data regarding these wellbeing impacts.

### Future Direction of the Research

The logical next step in this vein of research is to understand more about the identified themes, in particular those that are relatively novel to the literature, such as the social issues raised within this and other musical communities (e.g., relations across generations), the vital though declining role of musical groups, such as brass bands within local communities, and the potential for spiritual uplift through the group music making experience. Online surveys do not allow for the required level of depth for this form of theme exploration; the only way to gain more detailed information on these topics would be via structured interviews or focus groups. The conclusions from the present study would benefit from expansion on themes of interest by the use of these methods.

Whilst insights regarding the underlying psychophysiological drivers behind self-reported wellbeing effects (e.g., Fancourt et al., 2016) are beyond the remit of the present study, the present model offers future studies from this area a new avenue for research exploration. Having delineated a wide range of wellbeing impacts and depicted them in a model, studies can seek to test the theory that certain wellbeing impacts may be linked to particular underlying biomarkers. For example, we may question why some people in our study reported wellbeing benefits from the stress provided by competition whereas others viewed this as a negative aspect of group music-making. This avenue of research has the potential to identify specific pathways to wellbeing rather than pursue a general theory as to why music making helps us feel good. Another possibility is that personal wellbeing profiles may be drawn up for individuals that best reflect their particular drivers to wellbeing, whether they are physical, psychological, emotional, spiritual or social in nature. This advance in methodology toward personal profiling is in line with recent advances and understanding in relation to personalized medicine (Dzau et al., 2015).

### Conclusion

This paper has revealed how brass banding influences physical, social, psychological, emotional and spiritual resources, key elements of wellbeing as identified by both academic research and policy makers. Unbiased questioning meant we derived both positive and negative reports, thereby providing a balanced holistic view that has drawn out the unique pressures and challenges for brass band players, alongside the benefits that attract individuals of all ages to perform in their communities and competitions, in most cases for over 20 years. Our research has replicated a wide pattern of wellbeing benefits seen in choral practice that can now be taken forward as the basis of a model for general group music making benefits. The unique benefits and challenges of brass banding we have identified will directly inform the education and wellbeing support offered to these organizations in the future.

## Ethics Statement

This study was carried out in accordance with the recommendations of the University of Sheffield Ethics Committee with written informed consent from all subjects. All subjects gave written informed consent in accordance with the Declaration of Helsinki. The protocol was approved by the Ethics Committee at the Music Department, University of Sheffield.

## Author Contributions

VW and MB designed the study, created the materials, and took part jointly in meetings to discuss findings. MB conducted the primary analysis role with VW acting as second coder/reviewer. VW wrote the paper with MB offering review input.

### Conflict of Interest Statement

The authors declare that the research was conducted in the absence of any commercial or financial relationships that could be construed as a potential conflict of interest.

## Footnotes

^1^http://www.ibew.org.uk/How%20Many%20Brass%20Bands.pdf

^2^https://voicesnow.org.uk/research/big-choral-census/

^3^https://www.gov.uk/government/publications/five-ways-to-mental-wellbein

## Appendix 1

### Online Survey—Open Question

**How Does Brass Banding Affect Your Life**?

We would like to know how playing in brass bands has affected your life. Under each category below please tell us about any effects that you attribute to brass band activities. Activities can include anything related to brass bands, from solo preparation and practice work through to performances and community engagement. If you do not experience any effects of brass banding in relation to some or all of the categories below then please write

“No effect for me.”

Physical Effects:

Psychological Effects:

Social Effects:

Emotional Effects:

Spiritual Effects:

## Appendix 2

### Valence Disagreement Reports by Wellbeing Category

Spiritual: 1 U vs. +

Emotional: 2 U vs. +; 2 U vs. –

Social: 4 +/– vs. +; 6 U vs. +; 7 U vs. –; 2 +/– vs. –

Psychological: 3 +/– vs. +; 2 +/- vs. –

Physical: 4 +/– vs. +; 5 U vs. +; 2 U vs. –; 4 +/– vs. –
